# An Overview of Systematic Reviews of Chinese Herbal Medicine in the Treatment of Migraines

**DOI:** 10.3389/fphar.2022.924994

**Published:** 2022-07-25

**Authors:** Guojing Fu, Xueming Fan, Xiao Liang, Jingjing Wei, Min Jia, Shaojiao Liu, Wei Shen, Yunling Zhang

**Affiliations:** ^1^ Xiyuan Hospital of China Academy of Chinese Medical Sciences, Beijing, China; ^2^ Graduate School, Beijing University of Chinese Medicine, Beijing, China

**Keywords:** Chinese herbal medicine, migraine, overview, AMSTAR 2, PRISMA, grade

## Abstract

**Background:** In the past, systematic reviews (SRs) and meta-analyses (MAs) have been used to assess the efficacy of Chinese herbal medicine (CHM) in the treatment of migraines. However, robust conclusions have not yet been determined because of variations in the methodological and evidence quality of these SRs/MAs.

**Objectives:** We aimed to assess the methodological and reporting quality of SRs/MAs and evaluate the available evidence of the efficacy of CHM treatment of migraines.

**Methods:** We searched eight electronic databases from inception until 10 January 2022, without language restrictions. Two researchers were independently responsible for study screening and data extraction. The methodological and reporting quality of SRs/MAs were assessed using A Measurement Tool to Assess Systematic Reviews (AMSTAR) 2 and Preferred Reporting Items for Systematic Reviews and Meta-analyses (PRISMA). The evidence quality of included SRs/MAs was evaluated by Grading of Recommendations Assessment, Development and Evaluation (GRADE). In addition, a descriptive analysis of the included SRs/MAs was included.

**Results:** Sixteen SRs/MAs, including 69 outcomes, were finally included in this overview. Data synthesis of the included SRs/MAs outcomes showed that CHM plus Western medicine (WM) was beneficial in the improvement of migraines. In comparison, there was conflicting evidence for the effectiveness of CHM used alone. CHM was better than WM in improving responder rate and acute medication usage and was superior to placebo in improving migraine days, responder rate, and migraine duration. However, there was insufficient evidence to verify the effectiveness of CHM for migraine treatment regarding pain severity and migraine frequency. All the included SRs/MAs showed extremely low methodological and reporting quality. The results of the GRADE system indicated that the quality of most of the pooled evidence was very low.

**Conclusions:** CHM may be beneficial in improving migraines and can be used as a complementary therapy. However, we should treat the conclusions of the evaluated SRs/MAs cautiously because of the low quality of evidence. Future SRs/MAs should focus on improving methodological and reporting quality. High-quality randomized controlled trials (RCTs) are needed to provide strong evidence for the efficacy of CHM treatment of migraines.

## 1 Introduction

A migraine is typically a recurrent moderate-to-severe headache (Burch, 2019) and is one of the most prevalent and disabling disorders in the world. In the Global Burden of Disease Study 2016, migraines accounted for 45.1 million years of life lived with disability globally (GBD 2016 Neurology Collaborators, 2018; Dodick, 2018).

A recent study showed that migraines might be a risk factor for structural changes in the brain (Bashir et al., 2013). Drug therapy for migraines includes acute and preventive treatment. Acute treatment relieves the pain of a headache attack, and first-line treatments include non-steroidal anti-inflammatory drugs (NSAIDs, such as ibuprofen, diclofenac, aspirin, and naproxen), triptans, and ergot derivatives (Orr et al., 2015; Hsu et al., 2017; American Headache Society, 2018; Kouremenos et al., 2019; Steiner et al., 2019). Preventive therapy is available for patients suffering from mild to moderate migraines. The most widely accepted first-line treatments include anti-epileptic drugs (such as divalproex sodium, valproate sodium, and topiramate) and beta-blockers (such as metoprolol, propranolol, and timolol) (Huang et al., 2017; American Headache Society, 2018; Kouremenos et al., 2019; Kowacs et al., 2019). Many studies have validated the effectiveness of these recommended treatments. However, poor tolerability, varying degrees of adverse reactions, and contraindications limit the use of these treatments (Dodick, 2018; Burch, 2019; Steiner et al., 2019).

Chinese herbal medicine (CHM) is a popular treatment in complementary and alternative medicine (CAM), typically taking the form of herbal formulations consisting of a mixture of herbs. The clinical application of CHM is based on the syndrome type of the disease. CHM has been extensively employed for migraine treatment in China. The route of administration to treat migraines is mainly oral, and common dosage forms include decoctions, pills, powders, pastes, tablets, and granule formulations. CHM can treat migraines through multiple components, targets, and pathways, which may be related to four aspects as follows. First, by adjusting the level of neurotransmitters, such as regulating 5-hydroxytryptamine (5-HT) content and the expression of its receptor, increasing norepinephrine (NE) and dopamine (DA) release in the central catecholamine system, and regulating the release of human beta-endorphin (β-EP). Second, by regulating the levels of inflammatory factors, such as calcitonin gene-related peptide (CGRP), substance P (SP), homocysteine (Hcy), and interleukin (IL), to reduce neurogenic inflammation. Third, by boosting vasomotion to enhance cerebral blood flow, followed by improved hemorheology. Finally, by enhancing pain thresholds to alleviate or avoid headaches (Hou et al., 2017; Huang et al., 2020; Li et al., 2020; Chen et al., 2021; Wen et al., 2021). In the past, many evidence-based systematic reviews (SRs) or meta-analyses (MAs) have been performed to evaluate the use of CHM to treat migraines. Nevertheless, the quality of these SRs/MAs has not yet been assessed, and their conclusions were inconsistent. Consequently, the guidance for clinical users and physicians was limited.

Overviews are a relatively novel approach to evaluate the research methods and evidence from related SRs (Hunt et al., 2018). A Measurement Tool to Assess Systematic Reviews (AMSTAR) 2 and Preferred Reporting Items for Systematic Reviews and Meta-analyses (PRISMA) are usually employed to evaluate the methodological and reporting quality of SRs and MAs, respectively. Grading of Recommendations Assessment, Development and Evaluation (GRADE) is used to evaluate the quality of pooled evidence in SRs/MAs, reflecting the methodological quality of the original randomized controlled trials (RCTs) included in SRs/MAs. Consequently, overviews can present the overall quality of evidence relating to the use of CHM to treat migraines as well as the shortcomings of available research studies, enhancing the usefulness of existing evidence (Tian, 2019). In the present study, we performed an overview to critically evaluate the methodological and reporting quality of SRs/MAs relating to the use of CHM to treat migraines; we also assessed the current evidence for the use of CHM in treating migraines.

## 2 Methods

### 2.1 Search Strategy

Two investigators (GF and XF) independently searched the PubMed, Embase, Cochrane Library, Web of Science, China National Knowledge Infrastructure, Wan Fang Database of China, VIP Journals, and China Biomedical Literature databases from inception until 10 January 2022, without language restrictions. The search terms used were: “Migraine”; “Migraine Disorders”; “Migraine Headache”; “Acute Confusional Migraine”; “Migraine Variant”; “Sick Headache”; “Migraine Variant”; “Status Hemicranicus”; “Medicine, Chinese Traditional”; “Traditional Chinese Medicine”; “Herbal Medicine”; “Medicine, Traditional”; “Meta-Analysis”; “Data Pooling”; “Clinical Trial Overview”; and “Systematic Review”. The search strategy was adjusted according to the characteristics of each database. The details of the search strategy are shown in Table 1.

**TABLE 1:** The search strategy.

Search strategy for PubMed	
Number	Search terms
#1	“Migraine Disorders"[Mesh]
#2	migraine disorders[Title/Abstract]
#3	(((((((((((((((((((((((((((((((((((((((Disorder, Migraine[Title/Abstract]) OR Disorders, Migraine[Title/Abstract]) OR Migraine Disorder[Title/Abstract]) OR Migraine[Title/Abstract]) OR Migraines[Title/Abstract]) OR Migraine Headache[Title/Abstract]) OR Headache, Migraine[Title/Abstract]) OR Headaches, Migraine[Title/Abstract]) OR Migraine Headaches[Title/Abstract]) OR Acute Confusional Migraine[Title/Abstract]) OR Acute Confusional Migraines[Title/Abstract]) OR Migraine, Acute Confusional[Title/Abstract]) OR Migraines, Acute Confusional[Title/Abstract]) OR Status Migrainosus[Title/Abstract]) OR Hemicrania Migraine[Title/Abstract]) OR Hemicrania Migraines[Title/Abstract]) OR Migraine, Hemicrania[Title/Abstract]) OR Migraines, Hemicrania[Title/Abstract]) OR Migraine Variant[Title/Abstract]) OR Migraine Variants[Title/Abstract]) OR Variant, Migraine[Title/Abstract]) OR Variants, Migraine[Title/Abstract]) OR Sick Headache[Title/Abstract]) OR Headache, Sick[Title/Abstract]) OR Headaches, Sick[Title/Abstract]) OR Sick Headaches[Title/Abstract]) OR Abdominal Migraine[Title/Abstract]) OR Abdominal Migraines[Title/Abstract]) OR Migraine, Abdominal[Title/Abstract]) OR Migraines, Abdominal[Title/Abstract]) OR Cervical Migraine Syndrome[Title/Abstract]) OR Cervical Migraine Syndromes[Title/Abstract]) OR Migraine Syndrome, Cervical[Title/Abstract]) OR Migraine Syndromes, Cervical[Title/Abstract]) OR familial migraine[Title/Abstract]) OR headache, migrainous[Title/Abstract]) OR hemicrania[Title/Abstract]) OR status hemicranicus[Title/Abstract]))
#4	#1 OR #2 OR #3
#5	Medicine, chinese traditional[MeSH]
#6	((((((((((((((Medicine, Chinese Traditional[Title/Abstract]) OR (Traditional Chinese Medicine[Title/Abstract])) OR (Chung I Hsueh[Title/Abstract])) OR (Hsueh, Chung I[Title/Abstract])) OR (Traditional Medicine, Chinese[Title/Abstract])) OR (Zhong Yi Xue[Title/Abstract])) OR (Chinese Traditional Medicine[Title/Abstract])) OR (Chinese Medicine, Traditional[Title/Abstract])) OR (Traditional Tongue Diagnosis[Title/Abstract])) OR (Tongue Diagnoses, Traditional[Title/Abstract])) OR (Tongue Diagnosis, Traditional[Title/Abstract])) OR (Traditional Tongue Diagnoses[Title/Abstract])) OR (Traditional Tongue Assessment[Title/Abstract])) OR (Tongue Assessment, Traditional[Title/Abstract])) OR (Traditional Tongue Assessments[Title/Abstract])
#7	#5 OR #6
#8	Herbal Medicine[MeSH]
#9	(((((((((((Herbal Medicine[Title/Abstract]) OR (Medicine, Herbal[Title/Abstract])) OR (Hawaiian Herbal Medicine[Title/Abstract])) OR (Hawaiian Herbal Medicines[Title/Abstract])) OR (Herbal Medicine, Hawaiian[Title/Abstract])) OR (Herbal Medicines, Hawaiian[Title/Abstract])) OR (Medicine, Hawaiian Herbal[Title/Abstract])) OR (Medicines, Hawaiian Herbal[Title/Abstract])) OR (La’au Lapa’au[Title/Abstract])) OR (Laau Lapaau[Title/Abstract])) OR (La au Lapa au[Title/Abstract])) OR (Herbalism[Title/Abstract])
#10	#8 OR #9
#11	Medicine, Traditional[MeSH]
#12	((((((((((((((((Medicine, Traditional[Title/Abstract]) OR (Traditional Medicine[Title/Abstract])) OR (Home Remedies[Title/Abstract])) OR (Home Remedy[Title/Abstract])) OR (Remedies, Homeziy[Title/Abstract])) OR (Remedy, Home[Title/Abstract])) OR (Medicine, Primitive[Title/Abstract])) OR (Primitive Medicine[Title/Abstract])) OR (Medicine, Folk[Title/Abstract])) OR (Folk Medicine[Title/Abstract])) OR (Medicine, Indigenous[Title/Abstract])) OR (Indigenous Medicine[Title/Abstract])) OR (Folk Remedies[Title/Abstract])) OR (Folk Remedy[Title/Abstract])) OR (Remedies, Folk[Title/Abstract])) OR (Remedy, Folk[Title/Abstract])) OR (Ethnomedicine[Title/Abstract])
#13	#11 OR #12
#14	#7 OR #10 OR #13
#15	"Meta-Analysis" [Publication Type] OR "Meta-Analysis as Topic"[Mesh]
#16	(((((((((((((Meta-Analysis[Title/Abstract]) OR Meta-Analysis as Topic[Title/Abstract]) OR Data Pooling[Title/Abstract]) OR Data Poolings[Title/Abstract]) OR Overviews, Clinical Trial[Title/Abstract]) OR Clinical Trial Overviews[Title/Abstract]) OR Clinical Trial Overview[Title/Abstract]) OR Overview, Clinical Trial[Title/Abstract]) OR Cochrane review[Title/Abstract]) OR systematic review[Title/Abstract]) OR analysis, meta[Title/Abstract]) OR review, systematic[Title/Abstract]))
#17	#15 OR #16
#18	#4 AND #14 AND #17
The search strategy for Cochrane library	
Number	Search terms
#1	MeSH descriptor: [Migraine Disorders] explode all trees
#2	(Migraine Disorders OR Disorder, Migraine OR Disorders, Migraine OR Migraine Disorder OR Migraine OR Migraines OR Migraine Headache OR Headache, Migraine OR Acute Confusional Migraine OR Migraine Headaches OR Headaches, Migraine OR Acute Confusional Migraines OR Migraine, Acute Confusional OR Migraines, Acute Confusional OR Status Migrainosus OR Hemicrania Migraine):ti,ab,kw
#3	(Hemicrania Migraines OR Migraine, Hemicrania OR Migraines, Hemicrania OR Migraine Variant OR Migraine Variants OR Variant, Migraine OR Variants, Migraine OR Sick Headache OR Headache, Sick OR Headaches, Sick OR Sick Headaches OR Abdominal Migraine OR Abdominal Migraines OR Migraine, Abdominal OR Migraines, Abdominal OR headache, migrainous OR hemicrania OR status hemicranicus):ti,ab,kw
#4	#1 OR #2 OR #3
#5	MeSH descriptor: [Medicine, Chinese Traditional] explode all trees
#6	Traditional Chinese Medicine OR Chung I Hsueh OR Hsueh, Chung I OR Traditional Medicine, Chinese OR Zhong Yi Xue OR Chinese Traditional Medicine OR Chinese Medicine, Traditional OR Traditional Tongue Diagnosis OR Tongue Diagnoses, Traditional OR Tongue Diagnosis, Traditional OR Traditional Tongue Diagnoses OR Traditional Tongue Assessment OR Tongue Assessment, Traditional OR Traditional Tongue Assessments OR medicine, Chinese traditional OR Medicine, Chinese Traditional
#7	MeSH descriptor: [Herbal Medicine] explode all trees
#8	(Herbal Medicine OR Medicine, Herbal OR Hawaiian Herbal Medicine OR Hawaiian Herbal Medicines OR Herbal Medicine, Hawaiian OR Herbal Medicines, Hawaiian OR Medicine, Hawaiian Herbal OR Medicines, Hawaiian Herbal OR La’au Lapa’au OR Laau Lapaau OR La au Lapa au OR Herbalism):ti,ab,kw
#9	MeSH descriptor: [Medicine, Traditional] explode all trees
#10	(Medicine, Traditional OR Traditional Medicine OR Home Remedies OR Home Remedy OR Remedies, Homeziy OR Remedy, Home OR Medicine, Primitive OR Primitive Medicine OR Medicine, Folk OR Folk Medicine OR Medicine, Indigenous OR Indigenous Medicine OR Folk Remedies OR Folk Remedy OR Remedies, Folk OR Remedy, Folk OR Ethnomedicine):ti,ab,kw
#11	#5 OR #6 OR #7 OR #8 OR #9 OR #10
#12	MeSH descriptor: [Meta-Analysis as Topic] explode all trees
#13	(Meta-Analysis OR Meta-Analysis as Topic OR Data Pooling OR Data Poolings OR Overviews, Clinical Trial OR Clinical Trial Overviews OR Clinical Trial Overview OR Overview, Clinical Trial OR Cochrane review OR systematic review OR analysis, meta OR systematic review(topic) OR review, systematic):ti,ab,kw
#14	#12 OR #13
#15	#4 AND #11 AND #14
The search strategy for Embase	
Number	Search terms
#1	'migraine'/exp
#2	'disorder, migraine':ti,ab,kw OR 'migraine disorders':ti,ab,kw OR 'disorders, migraine':ti,ab,kw OR 'migraine disorder':ti,ab,kw OR migraines:ti,ab,kw OR 'migraine headache':ti,ab,kw OR 'headache, migraine':ti,ab,kw OR 'headaches, migraine':ti,ab,kw OR 'migraine headaches':ti,ab,kw OR 'acute confusional migraine':ti,ab,kw OR 'acute confusional migraines':ti,ab,kw OR 'migraine, acute confusional':ti,ab,kw OR 'migraines, acute confusional':ti,ab,kw OR 'status migrainosus':ti,ab,kw OR 'hemicrania migraine':ti,ab,kw OR 'hemicrania migraines':ti,ab,kw OR 'migraine, hemicrania':ti,ab,kw OR 'migraines, hemicrania':ti,ab,kw OR 'migraine variant':ti,ab,kw OR 'migraine variants':ti,ab,kw OR 'variant, migraine':ti,ab,kw OR 'variants, migraine':ti,ab,kw OR 'sick headache':ti,ab,kw OR 'headache, sick':ti,ab,kw OR 'headaches, sick':ti,ab,kw OR 'sick headaches':ti,ab,kw OR 'abdominal migraine':ti,ab,kw OR 'abdominal migraines':ti,ab,kw OR 'migraine, abdominal':ti,ab,kw OR 'migraines, abdominal':ti,ab,kw OR 'cervical migraine syndrome':ti,ab,kw OR 'cervical migraine syndromes':ti,ab,kw OR 'migraine syndrome, cervical':ti,ab,kw OR 'migraine syndromes, cervical':ti,ab,kw OR 'familial migraine':ti,ab,kw OR 'headache, migrainous':ti,ab,kw OR hemicrania:ti,ab,kw OR 'status hemicranicus':ti,ab,kw OR migraine:ti,ab,kw
#3	# 1 OR #2
#4	'chinese medicine'/exp
#5	'Chinese herbal medicine':ti,ab,kw OR 'medicine, Chinese traditional':ti,ab,kw OR 'traditional Chinese medicine':ti,ab,kw OR 'Chinese drug':ti,ab,kw OR 'Chinese medicinal formulas':ti,ab,kw OR 'fang ji fen lei':ti,ab,kw OR 'fang-ji-fen-lei':ti,ab,kw OR 'fangji fenlei':ti,ab,kw OR 'fangji-fenlei':ti,ab,kw OR 'fangjifenlei':ti,ab,kw OR 'traditional Chinese medicinal formula':ti,ab,kw OR 'traditional Chinese medicinal formulas':ti,ab,kw OR 'Chinese medicine':ti,ab,kw
#6	'traditional medicine'/exp
#7	'ethnomedicine':ti,ab,kw OR 'folk medicine':ti,ab,kw OR 'folk remedy':ti,ab,kw OR 'indigenous medicine':ti,ab,kw OR 'medicine, traditional':ti,ab,kw OR 'native healing':ti,ab,kw OR 'native medicine':ti,ab,kw OR 'traditional healing':ti,ab,kw OR 'traditional indigenous medicine':ti,ab,kw OR 'traditional medicine':ti,ab,kw
#8	'herbal medicine'/exp
#9	'botanical medicine':ti,ab,kw OR 'herb medicine':ti,ab,kw OR 'medicine, herbal':ti,ab,kw OR 'medicine, herbal':ti,ab,kw OR 'phyto-medicine':ti,ab,kw OR 'phytomedicine':ti,ab,kw OR 'plant medicine':ti,ab,kw OR 'plant-based medicine':ti,ab,kw OR 'herbal medicine':ti,ab,kw
#10	#4 OR #5 OR #6 OR #7 OR #8 OR #9
#11	'meta analysis'/exp OR 'meta analysis (topic)'/exp
#12	'systematic review' OR 'meta ananlysis' OR 'meta ananlysis(as topic)' OR 'systematic review(topic)' OR 'analysis, meta' OR 'meta analysis (topic)' OR 'review, systematic'
#13	#11 OR #12
#14	#3 AND #10 AND #13
Search strategy for Web of Science	
Number	Search terms
#1	TS=(disorder, migraine OR migraine disorders OR disorders, migraine OR migraine disorder OR migraines OR migraine headache OR headache, migraine OR headaches, migraine OR migraine headaches OR acute confusional migraine OR acute confusional migraines OR migraine, acute confusional OR migraines, acute confusional OR status migrainosus OR hemicrania migraine OR hemicrania migraines OR migraine, hemicrania OR migraines, hemicrania OR migraine variant OR migraine variants OR variant, migraine OR variants, migraine OR sick headache OR headache, sick OR headaches, sick OR sick headaches OR abdominal migraine OR abdominal migraines OR migraine, abdominal OR migraines, abdominal OR cervical migraine syndrome OR cervical migraine syndromes OR migraine syndrome, cervical OR migraine syndromes, cervical OR familial migraine OR headache, migrainous OR hemicrania OR status hemicranicus OR migraine
#2	TS=(Medicine, chinese traditional OR Traditional Chinese Medicine OR Chung I Hsueh OR Hsueh, Chung I OR Traditional Medicine, Chinese OR Zhong Yi Xue OR Chinese Traditional Medicine OR Chinese Medicine, Traditional OR Traditional Tongue Diagnosis OR Tongue Diagnoses, Traditional OR Tongue Diagnosis, Traditional OR Traditional Tongue Diagnoses OR Traditional Tongue Assessment OR Tongue Assessment, Traditional OR Traditional Tongue Assessments)
#3	TS=(Herbal Medicine OR Medicine, Herbal OR Hawaiian Herbal Medicine OR Hawaiian Herbal Medicines OR Herbal Medicine, Hawaiian OR Herbal Medicines, Hawaiian OR Medicine, Hawaiian Herbal OR Medicines, Hawaiian Herbal OR La'au Lapa'au OR Laau Lapaau OR La au Lapa au OR Herbalism)
#4	TS=(Medicine, Traditional OR Traditional Medicine OR Home Remedies OR Home Remedy OR Remedies, Homeziy OR Remedy, Home OR Medicine, Primitive OR Primitive Medicine OR Medicine, Folk OR Folk Medicine OR Medicine, Indigenous OR Indigenous Medicine OR Folk Remedies OR Folk Remedy OR Remedies, Folk OR Remedy, Folk OR Ethnomedicine)
#5	#2 OR #3 OR #4
#6	TS=(Meta-Analysis OR Meta-Analysis as Topic OR Data Pooling OR Data Poolings OR Overviews, Clinical Trial OR Clinical Trial Overviews OR Clinical Trial Overview OR Overview, Clinical Trial OR Cochrane review OR systematic review OR analysis, meta OR review, systematic)
#7	#1 AND #5 AND #6
**Search strategy for CNKI**	
(SU = '偏头痛' OR SU = '偏头风 ' OR SU = '偏正头风' OR SU = '头风' OR SU = '头痛' OR SU = '真头痛' OR SU = '厥头痛' OR SU = '半边头痛' OR SU = '首风' OR SU = '脑风' OR SU = '少阳头痛' OR SU = '疾首' OR SU = '风头痛' OR SU = '头角痛' OR SU = '头风病') AND (SU = '系统评价' OR SU = '荟萃分析 ' OR SU = 'Meta分析' OR SU = '系统综述' OR SU = '整合分析' OR SU = '数据分析' OR SU = '元分析')	
**Search strategy for Wanfang**	
主题:(("偏头痛" OR "偏头风" OR "偏正头风" OR "头风" OR "头痛" OR "真头痛" OR "厥头痛" OR "半边头痛" OR "首风" OR "脑风" OR "少阳头痛" OR "疾首"OR "风头痛" OR "头角痛" OR "头风病" ) AND ("系统评价" OR "荟萃分析" OR "Meta分析" OR "系统综述" OR "整合分析" OR "数据分析" OR "元分析" ))	
**Search strategy for VIP**	
((M=偏头痛 OR 偏头风 OR 偏正头风 OR 头风 OR 头痛 OR 真头痛 OR 厥头痛 OR 半边头痛 OR 首风 OR 脑风 OR 少阳头痛 OR 疾首 OR 风头痛 OR 头角痛 OR 头风病) OR (R=偏头痛 OR 偏头风 OR 偏正头风 OR 头风 OR 头痛 OR 真头痛 OR 厥头痛 OR 半边头痛 OR 首风 OR 脑风 OR 少阳头痛 OR 疾首 OR 风头痛 OR 头角痛 OR 头风病)) AND ((M=系统评价 OR 荟萃分析 OR Meta分析 OR 系统综述 OR 整合分析 OR 数据分析 OR 元分析) OR (R=系统评价 OR 荟萃分析 OR Meta分析 OR 系统综述 OR 整合分析 OR 数据分析 OR 元分析))	
**Search strategy for SinoMed**	
#1 "偏头痛"[常用字段:智能] OR "偏头风"[常用字段:智能] OR "偏正头风"[常用字段:智能] OR "头风"[常用字段:智能] OR "头痛"[常用字段:智能] OR "真头痛"[常用字段:智能] OR "厥头痛"[常用字段:智能] OR "半边头痛"[常用字段:智能] OR "首风"[常用字段:智能]#2 "脑风"[常用字段:智能] OR "少阳头痛"[常用字段:智能] OR "疾首"[常用字段:智能] OR "风头痛"[常用字段:智能] OR "头角痛"[常用字段:智能] OR "头风病"[常用字段:智能]#3 #1 OR #2 ("脑风"[常用字段:智能] OR "少阳头痛"[常用字段:智能] OR "疾首"[常用字段:智能] OR "风头痛"[常用字段:智能] OR "头角痛"[常用字段:智能] OR "头风病"[常用字段:智能]) OR ("偏头痛"[常用字段:智能] OR "偏头风"[常用字段:智能] OR "偏正头风"[常用字段:智能] OR "头风"[常用字段:智能] OR "头痛"[常用字段:智能] OR "真头痛"[常用字段:智能] OR "厥头痛"[常用字段:智能] OR "半边头痛"[常用字段:智能] OR "首风"[常用字段:智能])#4 "系统评价"[常用字段:智能] OR "荟萃分析"[常用字段:智能] OR "Meta分析"[常用字段:智能] OR "系统综述"[常用字段:智能] OR "整合分析"[常用字段:智能] OR "数据分析"[常用字段:智能] OR "元分析"[常用字段:智能]#5 #3 AND #4 (("脑风"[常用字段:智能] OR "少阳头痛"[常用字段:智能] OR "疾首"[常用字段:智能] OR "风头痛"[常用字段:智能] OR "头角痛"[常用字段:智能] OR "头风病"[常用字段:智能]) OR ("偏头痛"[常用字段:智能] OR "偏头风"[常用字段:智能] OR "偏正头风"[常用字段:智能] OR "头风"[常用字段:智能] OR "头痛"[常用字段:智能] OR "真头痛"[常用字段:智能] OR "厥头痛"[常用字段:智能] OR "半边头痛"[常用字段:智能] OR "首风"[常用字段:智能])) AND ("系统评价"[常用字段:智能] OR "荟萃分析"[常用字段:智能] OR "Meta分析"[常用字段:智能] OR "系统综述"[常用字段:智能] OR "整合分析"[常用字段:智能] OR "数据分析"[常用字段:智能] OR "元分析"[常用字段:智能])	

### 2.2 Eligibility Criteria

Each study met the following criteria:

• Type of study: SR/MA of RCTs related to the effectiveness of CHM for treating migraine.
• Type of participant: participants diagnosed with migraine by any internationally recognized classification, such as the diagnostic criteria for headache disorders, cranial neuralgias, and facial pain (ICHD-1) (Headache Classification Committee of the International Headache Society [IHS], 1988); The International Classification of Headache Disorders, 2nd edition (ICHD-2) (Headache Classification Committee of the International Headache Society [IHS], 2004); and The International Classification of Headache Disorders, 3rd edition (ICHD-3) (Headache Classification Committee of the International Headache Society [IHS], 2013).
• Type of intervention: the treatment group was treated with CHM formulas alone, combined with Western medicine, (WM) or placebo-controlled (PC), regardless of the drug form, dose, frequency, and duration of treatment. The comparison group was treated with WM or PC.
• Type of outcome: we referred to migraine clinical trial guidelines (Tassorelli et al., 2018) to develop outcome indicators, including migraine days, acute medication usage, responder rate, pain intensity (rated by measurement tools: visual analog scales [VAS] and/or numerical rating scales [NRS]), migraine frequency, migraine duration, adverse events, depression and anxiety, the patient’s reported outcome measures, and quality of life.

We used the following exclusion criteria: (1) the study was repeatedly published; (2) SRs/MAs with incomplete data or the evidence was not synthesized; (3) network meta-review; (4) the comparison group used CHM therapy; (5) the treatment group was treated with a single herbal medicine or herbal medicine ingredient.

### 2.3 Study Selection and Data Collection

Two researchers (GF and XF) were independently responsible for study screening and data extraction. Disagreements were resolved through discussion with a third researcher (WS). To preliminarily exclude the literature that was not relevant to the purpose of the study, initial screening was performed by reading titles and abstracts. We then carefully read the full text of papers that passed this screening to determine the final inclusion of studies. A data extraction form was designed and used to collect basic information about each study, including author information, year of publication, intervention measures, outcome indicators, study type, and conclusions.

### 2.4 Methodological Quality

AMSTAR-2 (Shea et al., 2017) was used to evaluate the methodological quality of the included SRs/MAs. This tool consists of 16 items and includes seven key items (2, 4, 7, 9, 11, 13, and 15). According to the judgment criteria of AMSTAR-2, each item was evaluated as “Yes”, “Partial Yes”, or “No”. SRs/MAs with no or only one non-critical item weakness were rated as high quality; more than one non-critical item weakness was rated as moderate quality; one critical flaw with or without non-critical item weakness was rated as low quality; and more than one critical flaw with or without non-critical item weaknesses was rated as extremely low quality. Two researchers (GF and XF) independently evaluated the methodological quality. Any disagreements were resolved with the help of a third researcher (WS).

### 2.5 Reporting Quality

The PRISMA statement was used to evaluate the reporting quality of the included SRs/MAs and included a 27-item checklist. Each item was evaluated as “Yes,” “Partial Yes,” or “No” based on seven domains: title, abstract, introduction, methods, results, discussion, and funding (Liberati et al., 2009; Moher et al., 2009). Two researchers (GF and XF) independently evaluated the reporting quality. Any disagreements were settled through dialogue with a third researcher (WS).

### 2.6 Quality of Evidence

The GRADE system was used to evaluate the quality of evidence, classifying a study into one of four levels: high, moderate, low, or very low. The evidence quality of included SRs/MAs was rated based on five factors: risk of bias, inconsistency, imprecision, indirectness, and publication bias. Two researchers (GF and XF) independently assessed the evidence quality. Any disagreements were resolved through discussion with a third researcher (WS).

### 2.7 Data Synthesis

A descriptive analysis of the included SRs/MAs was used in this overview. Risk ratios (RR), odds ratios (OR), 95% confidence intervals (CI), weighted mean differences (WMD), and standard mean differences (SMD) were included to summarize the outcomes. I^2^ was used to detect the heterogeneity of each included SR/MA.

## 3 Results

### 3.1 Literature Selection

**FIGURE 1:**
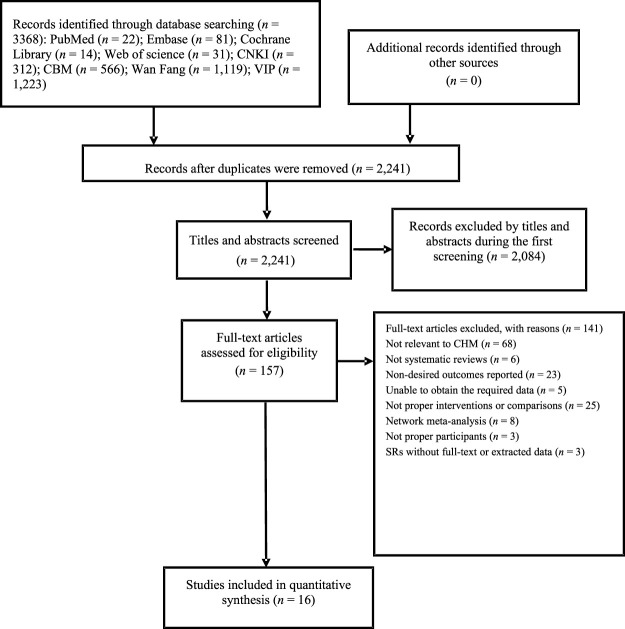
Flow diagram of the study process.

A total of 3368 items were discovered from the eight databases. NoteExpress 3.2.0 document manager was used for the literature selection. After the preliminary screening and elimination of duplicates, 157 articles were obtained for full-text reading. This overview ultimately included a total of 16 articles (Zhao et al., 2008; Chen et al., 2015; Xiao et al., 2015; Lai, 2017; Liu, 2018; Shan et al., 2018; Sun et al., 2018; Tian et al., 2018; Wang et al., 2019; Chen, 2020; Lei et al., 2020; Li et al., 2020; Lyu et al., 2020; Wu et al., 2020; Yu, 2020; Zhai et al., 2022). Figure 1 shows the entire study selection procedure.

### 3.2 Study Characteristics

Of the 16 SRs/MAs, 14 studies were published as journal articles, and two (Lai, 2017; Yu, 2020) were published as dissertations. These SRs/MAs were published between 2008 and 2022. Eleven SRs/MAs were published in Chinese and five (Xiao et al., 2015; Shan et al., 2018; Wang et al., 2019; Lyu et al., 2020; Wu et al., 2020) in English. The SRs/MAs all included migraine patients. Most SRs/MAs did not define the type of treatment, except one that described the type of intervention, which was preventive treatment (Lyu et al., 2020). Seven outcome measures were collected in 16 SRs/MAs, including migraine days, acute medication usage, responder rate, pain intensity, migraine frequency, migraine duration, and adverse events. The study characteristics are shown in Table 2.

**TABLE 2:** Systematic review/meta-analysis characteristics.

Study ID	Parti cipant	Acute/preventive treatment	Intervention	Control	Outcome measure	Number of RCTs (participants)	Design
Liu, 2018	Migraine	NR	Toutongning capsule	Flunarizine	④⑤	24/2572	RCT
Tian, 2018	Migraine	NR	Toutongning capsule plus flunarizine	Flunarizine	④⑤⑦	28/3001	RCT
Sun, 2018	Migraine	NR	Toutongning capsule plus flunarizine	Flunarizine	④	16/1726	RCT
Lei, 2020	Migraine	NR	Yangxue Qingnao granules or plus calcium channel blocker	Calcium channel blocker	④⑤⑥⑦	23/2308	RCT
Zhao, 2008	Migraine	NR	Yangxue Qingnao granules	Flunarizine	⑤⑥	8/707	RCT
Yu, 2020	Migraine	NR	Chuanxiong Qingnao granules or plus flunarizine	Flunarizine or PC	④⑤⑥⑦	17/1865	RCT
Chen,2020	Migraine	NR	Chuanxiong Qingnao granules plus WM	WM	④⑦	11/1099	RCT
Li, 2020	Migraine	NR	Chuanxiong Qingnao granules plus flunarizine	Flunarizine	④⑦	9/883	RCT
Chen,2015	Migraine	NR	Zhengtian pill or plus flunarizine	Flunarizine or PC	①④⑤⑥⑦	15/956	RCT
Zhai, 2022	Migraine	NR	Duliang Ruan capsules or plus WM/PC	WM or PC	④⑤⑥⑦	14/1325	RCT
Xiao, 2015	Episodic migraine	NR	CHM	PC	③④⑤	7/582	RCT
Wu, 2020	Migraine	NR	Sanpian decoction or plus Sibelium	Sibelium	④⑤⑦	15/1377	RCT
Wang, 2019	Migraine	NR	CXCT Chinese medicine decoction or plus WM	WM	④⑦	37/3307	RCT
Lyu, 2020	Episodic migraine	Preventive treatment	CHM	flunarizine	①③④⑤⑥②	35/2840	RCT
Shan, 2018	Migraine	NR	Herbal formulas that must include herb the Chuanxiong	PC or conventional pharmacotherapy	①④⑤⑥⑦	19/1832	RCT
Lai, 2017	Migraine	NR	CHM	Flunarizine	①④⑤⑥⑦	25/1904	RCT

NR, no report; WM, Western medicine (the SRs/MAs did not specify the name of the western drug); CHM, Chinese herbal medicine formulas (the SRs/MAs included a variety of herbal formulas); PC, placebo-controlled; RCT, randomized controlled trial.

Outcome measures, ①Migraine days; ②Acute medication usage; ③Responder rate; ④Pain intensity (including VAS scores, NRS scores); ⑤Migraine frequency; ⑥Migraine duration; ⑦Adverse events; ⑧Depression and anxiety; ⑨Patient’s reported outcome measures; ⑩Quality of life.

### 3.3 Description of Chinese Herbal Medicine

A total of 74 CHM formulas were found in these studies, including 64 decoctions and 10 patent medicines. All herbs appeared 671 times. The top seven most frequently used herbs were *Rhizoma Ligustici Chuanxiong* (64/9.54%), *Radix Angelicae Dahuricae* (38/5.66%), *Radix Paeoniae Alba* (27/4.02%), *Radix Glycyrrhizae Uralensis* (26/3.87%), *Radix Angelicae Sinensis* (25/3.73%), *Rhizoma Gastrodiae* (21/3.13%), and *Herba Asari* (21/3.13%). Table 3 shows the details of these seven herbs. The top three most frequently used CHM patent medicines were Yangxue Qingnao granules, Zhengtian pills, and Toutongning capsules. Details are provided in Table 4.

**TABLE 3:** Details of frequently used herbs in CHM formulas.

Chinese name	Pharmaceutical name	Species	Family	Frequency	Rate (%)
Chuanxiong	Rhizoma Ligustici	Ligusticum striatum DC	Apiaceae	64	9.54
	Chuanxiong				
Baizhi	Radix Angelicae Dahuricae	Angelica dahurica (Fisch. ex Hoffm.) Benth.et Hook. f. ex Franch. et Sav	Apiaceae	38	5.66
Baishao	Radix paeoniae alba	Paeonia lactiflora Pall	Paeoniaceae	27	4.02
Gancao	Radix glycyrrhizae uralensis	Glycyrrhiza uralensis Fisch. Ex DC	Fabaceae	26	3.87
Danggui	Radix Angelicae Sinensis	Angelica sinensis (Oliv.) Diels	Apiaceae	25	3.73
Tianma	Rhizoma gastrodiae	Gastrodia elata Bl	Orchidaceae	21	3.13
Xixin	Herba Asari	Asarum sieboldii Miq	Aristolochiaceae	21	3.13

**TABLE 4:** Composition of Chinese patent medicines used in the included studies.

Name of Chinese patent medicine	Source	Chinese name, pharmaceutical name, species, family	Quality control reported? (Y/N)
Yangxue Qingnao granules	Tasly Pharmaceutical Group Co., Ltd.	Danggui, Radix Angelicae Sinensis, Angelica sinensis (Oliv.) Diels, Apiaceae; Chanxiong, Rhizoma Ligustici Chuanxiong , Ligusticum striatum DC, Apiaceae Baishao, Radix Paeoniae Alba, Paeonia lactiflora Pall., Paeoniaceae; Shudihaung, Radix Rehmanniae Preparata, Rehmannia glutinosa Libosch, Scrophulariaceae Gouteng, Ramulus Uncariae cum Uncis, Uncaria rhynchophylla(Miq).Miq.ex Havil, Rubiaceae; Jixueteng, Caulis Spatholobi, Spatholobus suberectus Dunn, Leguminosae; Xiakucao, Spica Prunellae, Prunella vulgaris Linn, Labiatae; Juemingzi, Semen Cassiae, Cassia obtusifolia L., Leguminosae; Zhenzhumu, Concha Margaritifera, Hyriopsis cumingii (Lea) Yanhusuo, Rhizoma Corydalis, Corydalis yanhusuo W. T. Wang, Papaveraceae; Xixin, Herba Asari, Asarum sieboldii Miq., Aristolochiaceae	Y- Prepared according to the Pharmacopoeia of the People’s Republic of China; Z10960082
Zhengtian pill	China Resources Sanjiu Medical & Pharmaceutical Co., Ltd.	Chuanxiong, Rhizoma Ligustici Chuanxiong , Ligusticum striatum DC, Apiaceae Qianghuo, Rhizoma et Radix Notopterygii, Notopterygium incisum Ting ex H. T. Chang, Apiaceae; Fangfeng, Radix Saposhnikoviae, Saposhnikovia divaricate (Turcz. Ex Ledeb.) Schischk., Apiaceae; Baizhi, Radix Angelicae Dahuricae, Angelica dahurica (Fisch. ex Hoffm.) Benth. et Hook. f. ex Franch. et Sav, Apiaceae Gouteng, Ramulus Uncariae Cum Uncis, Uncaria rhynchophylla(Miq)Miq.ex Havil, Rubiaceae; Taoren, Semen Persicae, Prunus persica (L.) Batsch, Rosaceae; Honghua, Flos Carthami, Carthamus tinctorius L., Asteraceae; Danggui, Radix Angelicae Sinensis, Angelica sinensis (Oliv.) Diels, Apiaceae; Jixueteng, Caulis Spatholobi, Spatholobus suberectus Dunn, Leguminosae; Dihuang, Radix Rehmanniae Recens, Rehmannia glutinosa (Gaertn.) DC., Orobanchaceae; Duhuo, Radix Angelicae Pubescentis, Heracleum hemsleyanum Diels, Apiaceae; Fuzi, Radix Aconiti Lateralis Preparat, Aconitum armichaeli Debx, Ranunculaceae; Mahuang, Herba Ephedrae, Ephedra sinica Stapf, Ephedraceae; Xixin, Herba Asari, Asarum sieboldii Miq., Aristolochiaceae; Baishao, Radix Paeoniae Alba, Paeonia lactiflora Pall., Paeoniaceae	Y- Prepared according to the Pharmacopoeia of the People’s Republic of China; Z44020711
Buchang Toutong Ning capsules	Shanxi Buchang Pharmaceutical Co. LTD.	Tufuling, Rhizoma smilacis glabrae, Smilax glabra Roxb., Liliaceae; Tianma, Rhizoma gastrodiae, Gastrodia elata Bl., Orchidaceae; Zhiheshouwu, Radix polygoni multiflori preparata, Fallopia multiflora (Thunb.) Harald., Polygonaceae; Danggui, radix angelicae sinensis, Angelica sinensis (Oliv.) Diels, Apiaceae; Fangfeng, Radix Saposhnikoviae, Saposhnikovia divaricate (Turcz. Ex Ledeb.) Schischk., Apiaceae; Quanxie, Scorpio, Buthus martensii karsch	Y- Prepared according to the Pharmacopoeia of the People’s Republic of China; Z20026851
Chuanxiong Qingnao granules	Jichuan Pharmaceutical Group Co., Ltd.	Danggui, Radix Angelicae Sinensis, Angelica sinensis (Oliv.) Diels, Apiaceae; Chanxiong, Rhizoma Ligustici Chuanxiong , Ligusticum striatum DC, Apiaceae; Fangfeng, Radix Saposhnikoviae, Saposhnikovia divaricate (Turcz. Ex Ledeb.) Schischk., Apiaceae; Baizhi, Radix Angelicae Dahuricae, Angelica dahurica (Fisch. ex Hoffm.) Benth. et Hook. f. ex Franch. et Sav, Apiaceae; Maidong, Radix Ophiopogonis, root of Ophiopogon japonicus (Thunb.) Ker Gawl., Asparagaceae; Xixin, Herba Asari, Asarum sieboldii Miq., Aristolochiaceae; Qianghuo, Rhizoma et Radix Notopterygii,Notopterygium incisum Ting ex H. T. Chang, Apiaceae; Duhuo, Radix Angelicae Pubescentis, Heracleum hemsleyanum Diels, Apiaceae; Canzhu, Rhizoma atractylodis, Atractylodes lancea (Thunb.) DC.,Compositae; Juhua, Flos Chrysanthemi, Dendranthema morifolium (Ramat.) Tzvel., Compositae; Manjingzi, Fructus Viticis, Vitex trifolia L. var. simplicifolia Cham., Verbenaceae; Huangqin, Radix Scutellariae, Scutellaria baicalensis, Lamiaceae; Gancao, Radix Glycyrrhizae uralensis, Glycyrrhiza uralensis Fisch. Ex DC., Fabaceae; Shengjiang, Rhizoma Zingiberis Recens, Zingiber officinale Roscoe, Zingiberaceae	Y- Prepared according to the Pharmacopoeia of the People’s Republic of China; Z- Z20060177
Tongxinluo capsules	Shijiazhuang Yiling Pharmaceutical Co., Ltd.	Renshen, Ginseng Radix et Rhizoma, Panax ginseng C. A. Mey., Araliaceae; Shuizhi, body of Hirudo nipponia (Whitman); Quanxie, Scorpio, Buthus martensii karsch; Chishao, Radix Peoniae rubra, P. veitchii Lynch, Paeoniaceae; Chantui, Cryptotympana pustulata Fabricius; Tubiechong, body of Eupolyphaga sinensis Walker; Wugong, Scolopendra,Scolopendra subspinipes, Psittacidae; Tanxiang, Sandalwood, Santalum album Linn., Santalaceae; Jiangxiang, Dalbergiae odoriferae lignum, Dalbergia odorifera T. Chen, Fabaceae; Ruxiang, Olibanum, Boswellia carterii Birdw, Buseraceae; Suanzaoren, Ziziphi spinosae semen, Ziziphus jujuba Mill.var.spinosa (Bunge) Hu ex H.F.Chou, Rhamnaceae Bingpian, Borneolum, Dryobalanops aromatica Gaertn.f., Dipterocarpaceae	Y- Prepared according to the Pharmacopoeia of the People’s Republic of China; Z- Z19980015
Zhengtian capsules	China Resources Sanjiu Medical & Pharmaceutical Co., Ltd.	Chuanxiong, Rhizoma Ligustici Chuanxiong, Ligusticum striatum DC, Apiaceae Qianghuo, Rhizoma et Radix Notopterygii, Notopterygium incisum Ting ex H. T. Chang, Apiaceae; Fangfeng, Radix Saposhnikoviae, Saposhnikovia divaricate (Turcz. Ex Ledeb.) Schischk., Apiaceae; Baizhi, Radix Angelicae Dahuricae, Angelica dahurica (Fisch. ex Hoffm.) Benth. et Hook. f. ex Franch. et Sav, Apiaceae Gouteng, Ramulus Uncariae Cum Uncis, Uncaria rhynchophylla(Miq)Miq.ex Havil, Rubiaceae; Taoren, Semen Persicae, Prunus persica (L.) Batsch, Rosaceae; Honghua, Flos Carthami, Carthamus tinctorius L., Asteraceae; Danggui, Radix Angelicae Sinensis, Angelica sinensis (Oliv.) Diels, Apiaceae; Jixueteng, Caulis Spatholobi, Spatholobus suberectus Dunn, Leguminosae; Dihuang, Radix Rehmanniae Recens, Rehmannia glutinosa (Gaertn.) DC., Orobanchaceae; Duhuo, Radix Angelicae Pubescentis, Heracleum hemsleyanum Diels, Apiaceae; Fuzi, Radix Aconiti Lateralis Preparat, Aconitum armichaeli Debx, Ranunculaceae; Mahuang, Herba Ephedrae, Ephedra sinica Stapf, Ephedraceae; Xixin, Herba Asari, Asarum sieboldii Miq., Aristolochiaceae; Baishao, Radix Paeoniae Alba, Paeonia lactiflora Pall., Paeoniaceae	Y- Prepared according to the Pharmacopoeia of the People’s Republic of China; Z20010142
Danzhen Toutong capsules	Qinghai Yixin Pharmaceutical Co., Ltd.	Danshen, Radix Salvia Miltiorrhizae, Salvia miltiorrhiza Bunge, Lamiaceae; Xiakucao, Spica Prunellae, Prunella vulgaris Linn, Labiatae; Chuanxiong, Rhizoma Ligustici Chuanxiong , Ligusticum striatum DC, Apiaceae Danggui, radix angelicae sinensis, Angelica sinensis (Oliv.) Diels, Apiaceae; Baishao, Radix Paeoniae Alba, Paeonia lactiflora Pall., Paeoniaceae; Shengdihuang, Radix Rehmanniae Preparata, root of Rehmannia glutinosa (Gaertn.) DC., Orobanchaceae; Zhenzhumu, Concha Margaritifera, Hyriopsis cumingii (Lea) Jixueteng, Caulis Spatholobi, Spatholobus suberectus Dunn, Leguminosae; Juhua, Flos Chrysanthemi, Dendranthema morifolium (Ramat.) Tzvel., Compositae; Jili, Fructus Tribuli, Tribulus terrester Linn., Zygophyllaceae; Gouteng, Ramulus Uncariae Cum Uncis, Uncaria rhynchophylla(Miq)Miq.ex Havil, Rubiaceae; Xixin, Herba Asari, Asarum Sieboldii Miq., Aristolochiaceae	Y- Prepared according to the Pharmacopoeia of the People’s Republic of China; Z10950004
Tianshu capsules	Jiangsu Kangyuan Meiyu Biological Medicine Co., Ltd.	Chuanxiong, Rhizoma Ligustici Chuanxiong , Ligusticum striatum DC, Apiaceae Tianma, Rhizoma Gastrodiae, Gastrodia elata Bl., Orchidaceae;	Y- Prepared according to the Pharmacopoeia of the People’s Republic of China; Z20025871
Fufang Yangjiao granules	Beijing Tongrentang Natural Medicine (Tangshan) Co., LTD.	Yangjiao, Cornu Saigae Tataricae, Bos taurus domesticus gmelin, Bovidae; Chuanxiong, Rhizoma Ligustici Chuanxiong , Ligusticum striatum DC, Apiaceae Baizhi, Radix Angelicae Dahuricae, Angelica dahurica (Fisch. ex Hoffm.) Benth. et Hook. f. ex Franch. et Sav,Apiaceae; Chuanwu, Radix Aconiti, Aconitum carmichaeli Debx., Ranunculaceae	Y- Prepared according to the Pharmacopoeia of the People’s Republic of China; Z22022995
Duliangruan capsules	Chongqing Huasen Pharmaceutical Co. LTD.	Chanxiong, Rhizoma Ligustici Chuanxiong , Ligusticum striatum DC, Apiaceae Baizhi, Radix Angelicae Dahuricae, Angelica dahurica (Fisch. ex Hoffm.) Benth. et Hook. f. ex Franch. et Sav,Apiaceae	Y- Prepared according to the Pharmacopoeia of the People’s Republic of China; Z20055185

### 3.4 Methodological Quality

We evaluated the methodological quality of 16 SRs/MAs. As shown in Table 5, the methodological quality of all the included SRs/MAs was extremely low. For the evaluation of key items, the protocol was rarely registered before the commencement of the review and was only registered in one SR/MA (Lyu et al., 2020). None of the SRs/MAs offered a list of excluded studies or explained their exclusion. Eight SRs/MAs were appraised as “No” for key item 11 because the necessary statistical approaches for substantial heterogeneity were not applied. When analyzing and discussing the outcomes of their reviews, six SRs/MAs (Liu, 2018; Sun et al., 2018; Tian et al., 2018; Chen, 2020; Li et al., 2020; Zhai et al., 2022) did not emphasize assessing the risk of bias.

**TABLE 5:** Results of the AMSTAR 2 assessment.

Study ID	AMSTAR-2 items																Overall quality
	1	2	3	4	5	6	7	8	9	10	11	12	13	14	15	16	
Liu, 2018	Y	N	Y	PY	Y	N	N	N	PY	N	N	N	N	N	N	N	Extremely low
Tian, 2018	Y	N	Y	PY	Y	N	N	PY	PY	N	N	N	N	N	N	N	Extremely low
Sun, 2018	Y	N	Y	PY	N	Y	N	PY	PY	N	N	N	N	Y	Y	N	Extremely low
Lei, 2020	Y	N	Y	PY	Y	Y	N	PY	Y	N	Y	Y	Y	Y	Y	N	Extremely low
Zhao, 2008	Y	N	Y	N	N	N	N	PY	PY	N	N	N	Y	Y	N	N	Extremely low
Yu, 2020	Y	N	Y	PY	Y	Y	N	PY	Y	N	Y	N	Y	Y	N	N	Extremely low
Chen, 2020	Y	N	Y	PY	N	Y	N	PY	Y	N	N	N	N	Y	N	N	Extremely low
Li, 2020	Y	N	Y	PY	Y	Y	N	PY	PY	N	N	N	N	N	N	N	Extremely low
Chen, 2015	Y	N	Y	PY	Y	Y	N	PY	PY	N	N	N	Y	N	N	N	Extremely low
Zhai, 2022	Y	N	Y	PY	Y	Y	N	PY	Y	N	Y	N	N	Y	N	N	Extremely low
Xiao, 2015	Y	N	Y	PY	N	Y	N	Y	Y	N	Y	N	Y	Y	N	Y	Extremely low
Wu, 2020	Y	N	Y	PY	N	N	N	PY	PY	N	N	N	Y	N	N	Y	Extremely low
Wang, 2019	Y	N	Y	PY	N	Y	N	PY	Y	N	Y	N	Y	Y	Y	Y	Extremely low
Lai, 2017	Y	N	Y	PY	Y	Y	N	PY	Y	N	Y	N	Y	Y	N	N	Extremely low
Lyu, 2020	Y	PY	Y	PY	Y	Y	N	PY	Y	N	Y	Y	Y	Y	Y	Y	Extremely low
Shan, 2018	Y	PY	Y	PY	Y	Y	N	Y	Y	N	Y	N	Y	N	Y	Y	Extremely low

N, no; PY, partially yes; Y, yes.

### 3.5 Reporting Quality

As shown in Table 6, 20 of 27 items were well-reported, with a response rate of “Yes” or “Partial Yes” exceeding 80%. However, 7 of these 27 items were inadequately reported, especially for item 5 (Protocol and registration), item 8 (Search), and item 27 (Funding), with a less than 50% response rate of “Yes” and “Partial Yes.” Only one SR/MA (Lyu et al., 2020) was registered before the review began. Two SRs/MAs (Lei et al., 2020; Yu, 2020) presented an electronic search approach for at least one database. Two SRs/MAs (Wang et al., 2019; Lyu et al., 2020) described the sources of funding for the SR/MAs and described the role of funders in the systematic review.

**TABLE 6:** Results of the PRISMA assessment.

Section/Topic	Items	Liu, 2018	Tian, 2018	Sun, 2018	Lei, 2020	Zhao, 2008	Yu, 2020	Chen, 2020	Li, 2020	Chen, 2015	Zhai, 2022	Xiao, 2015	Wu,2020	Shan,2018	Wang,2019	Lai,2017	Lyu,2020	Number of yes and partially yes(%)
Title	1.Title	Y	Y	Y	Y	Y	Y	Y	Y	Y	Y	Y	Y	Y	Y	Y	Y	16(100%)
Abstract	2.Structured summary	PY	PY	PY	PY	PY	PY	PY	PY	PY	PY	PY	PY	PY	PY	PY	PY	16(100%)
Introduction	3.Rationale	Y	Y	Y	Y	Y	Y	Y	Y	Y	Y	Y	Y	Y	Y	Y	Y	16(100%)
	4.Objectives	Y	Y	Y	Y	Y	Y	Y	Y	Y	Y	Y	Y	Y	Y	Y	Y	16(100%)
	5.Protocol and registration	N	N	N	N	N	N	N	N	N	N	N	N	N	N	N	Y	1(6.25%)
Methods	6.Eligibility criteria	Y	Y	Y	Y	Y	Y	Y	Y	Y	Y	Y	Y	Y	Y	Y	Y	16(100%)
	7.Information sources	PY	PY	PY	PY	PY	PY	PY	PY	PY	PY	PY	PY	PY	PY	PY	PY	16(100%)
	8.Search	N	N	N	Y	N	Y	N	N	N	N	N	N	N	N	N	N	2(12.50%)
	9.Study selection	Y	Y	N	Y	N	Y	N	Y	PY	Y	N	N	Y	N	Y	Y	10(62.5%)
	10.Data collection process	N	N	N	Y	N	Y	Y	Y	Y	Y	Y	N	Y	Y	Y	Y	11(68.75%)
	11.Data items	PY	N	PY	PY	N	PY	N	PY	PY	PY	PY	PY	Y	PY	PY	PY	13(81.25%)
	12.Risk of bias in individual studies	Y	Y	Y	Y	Y	Y	Y	Y	Y	Y	Y	Y	Y	Y	Y	Y	16(100%)
	13.Summary measures	Y	Y	Y	Y	Y	Y	Y	Y	Y	Y	Y	Y	Y	Y	Y	Y	16(100%)
	14.Synthesis of results	Y	Y	Y	Y	Y	Y	Y	Y	Y	Y	Y	Y	Y	Y	Y	Y	16(100%)
	15.Risk of bias across studies	Y	Y	Y	Y	N	Y	Y	N	Y	Y	Y	Y	Y	Y	Y	Y	14(87.50%)
	16.Additional analyses	N	Y	Y	Y	Y	Y	Y	N	Y	Y	N	N	Y	N	Y	Y	11(68.75%)
Results	17.Study selection	PY	Y	PY	Y	Y	Y	Y	Y	Y	Y	N	Y	Y	Y	Y	Y	15(93.75%)
	18.Study characteristics	N	Y	Y	Y	PY	Y	PY	Y	Y	Y	Y	Y	Y	Y	Y	Y	15(93.75%)
	19.Risk of bias within studies	N	Y	Y	Y	Y	Y	Y	Y	PY	Y	Y	Y	Y	Y	Y	Y	15(93.75%)
	20.Results of individual studies	Y	Y	Y	Y	Y	Y	Y	Y	Y	Y	Y	Y	Y	Y	Y	Y	16(100%)
	21.Synthesis of results	Y	Y	Y	Y	N	Y	Y	Y	Y	Y	Y	Y	Y	Y	Y	Y	16(100%)
	22.Risk of bias across studies	Y	Y	Y	Y	N	Y	Y	N	Y	Y	N	Y	Y	Y	Y	Y	13(81.25%)
	23.Additional analyses	N	N	Y	Y	N	Y	N	N	Y	Y	Y	N	N	N	Y	Y	8(50%)
Discussion	24.Summary of evidence	PY	PY	PY	PY	PY	PY	PY	PY	PY	PY	PY	PY	PY	PY	PY	PY	16(100%)
	25.Limitations	PY	Y	Y	Y	PY	Y	Y	Y	Y	Y	Y	Y	PY	PY	Y	Y	16(100%)
	26.Conclusions	Y	Y	Y	Y	Y	Y	Y	Y	Y	Y	Y	Y	Y	Y	Y	Y	16(100%)
Funding	27.Funding	N	N	N	N	N	N	N	N	N	N	N	N	N	Y	N	Y	2(12.50%)

### 3.6 Effects of Chinese Herbal Medicine Interventions

#### 3.6.1 Migraine Days

##### 3.6.1.1 Chinese Herbal Medicine vs. Placebo-Controlled

Two SRs/MAs (Chen et al., 2015; Shan et al., 2018) reported migraine days, and the results demonstrated that CHM was superior to PC in improving migraine days.

##### 3.6.1.2 Chinese Herbal Medicines vs. Western Medicines

Migraine days were assessed in three SRs/MAs (Lai, 2017; Shan et al., 2018; Lyu et al., 2020), but did not arrive at consistent conclusions: two showed that CHM was similar to WM, while one showed CHM was better than WM in reducing migraine days.

#### 3.6.2 Responder Rate

Responder rate was assessed in two SRs/MAs (Xiao et al., 2015; Lyu et al., 2020): one SR (Lyu et al., 2020) indicated that CHM improved responder rate compared with WM; another SR (Xiao et al., 2015) also showed that CHM was better than PC.

#### 3.6.3 Pain Intensity

##### 3.6.3.1 Chinese Herbal Medicine vs. Placebo-Controlled

Pain intensity was evaluated in four SRs/MAs (Chen et al., 2015; Xiao et al., 2015; Shan et al., 2018; Yu, 2020). Three SRs/MAs (Chen et al., 2015; Xiao et al., 2015; Shan et al., 2018) concluded that CHM was better than PC in terms of pain intensity.

##### 3.6.3.2 Chinese Herbal Medicines vs. Western Medicines

Six SRs/MAs (Lai, 2017; Liu, 2018; Lyu et al., 2020; Wu et al., 2020; Yu, 2020; Zhai et al., 2022) reported pain intensity, and three of them evaluated VAS. Of these three, two SRs/MAs (Liu, 2018; Wu et al., 2020) indicated that CHM was inferior to WM in reducing headache intensity. One SR (Lyu et al., 2020) was performed for a subgroup analysis based on treatment duration. In the SR by Lyu et al. (2020), CHM was found to be better than WM in reducing headache intensity at the end of treatment, while there was no significant difference at the end of follow-up. One SR (Yu, 2020) evaluated pain intensity by NRS, which showed CHM was similar to WM at improving pain intensity. Three SRs/MAs (Lai, 2017; Shan et al., 2018; Zhai et al., 2022) reported pain intensity: two (Lai, 2017; Shan et al., 2018) showed that CHM noticeably reduced pain intensity compared with WM, while no statistical difference was found in the study by Zhai et al. (2022).

##### 3.6.3.3 Chinese Herbal Medicine Plus Western Medicines vs. Western Medicines

Eight SRs/MAs (Sun et al., 2018; Tian et al., 2018; Wang et al., 2019; Chen, 2020; Lei et al., 2020; Li et al., 2020; Yu, 2020; Zhai et al., 2022) evaluated pain intensity of CHM plus WM; seven of these articles measured VAS, and two SR (Lai, 2017; Zhai et al., 2022) combined VAS and NRS scores. All showed that CHM plus WM had a better effect in reducing pain intensity than WM alone.

#### 3.6.4 Migraine Frequency

##### 3.6.4.1 Chinese Herbal Medicine vs. Placebo-Controlled

Migraine frequency was evaluated in four SRs/MAs (Chen et al., 2015; Xiao et al., 2015; Shan et al., 2018; Yu, 2020). Three of these (Chen et al., 2015; Xiao et al., 2015; Shan et al., 2018) showed that CHM was superior to PC in reducing migraine frequency. One SR (Yu, 2020), including 438 participants in two RCTs, indicated that CHM and WM were equally effective.

##### 3.6.4.2 Chinese Herbal Medicines vs. Western Medicines

Eight SRs/MAs (Zhao et al., 2008; Lai, 2017; Liu, 2018; Shan et al., 2018; Lyu et al., 2020; Wu et al., 2020; Yu, 2020; Zhai et al., 2022) assessed migraine frequency, while only one SR (Lai, 2017) described units of migraine frequency, including the number of attacks per month and headache attack score. Except for in one SR (Yu, 2020), these SRs/MAs showed that CHM was more effective than WM alone in improving migraine frequency.

##### 3.6.4.3 Chinese Herbal Medicine Plus Western Medicines vs. Western Medicines

Three SRs/MAs (Tian et al., 2018; Lei et al., 2020; Zhai et al., 2022) investigated the effectiveness of CHM plus WM on migraine frequency. A meta-analysis of these SRs/MAs found that CHM plus WM significantly improved migraine frequency compared with WM alone.

#### 3.6.5 Migraine Duration

##### 3.6.5.1 Chinese Herbal Medicine vs. Placebo-Controlled

Two SRs/MAs (Chen et al., 2015; Shan et al., 2018) indicated CHM significantly improved migraine duration compared with PC.

##### 3.6.5.2 Chinese Herbal Medicines vs. Western Medicines

Migraine duration was assessed in six SRs/MAs (Zhao et al., 2008; Lai, 2017; Shan et al., 2018; Lyu et al., 2020; Yu, 2020; Zhai et al., 2022). Three SRs/MAs showed that CHM was superior to WM in reducing migraine duration (Lai, 2017; Shan et al., 2018; Zhai et al., 2022), whereas the results of the meta-analysis were inconsistent in the studies by Yu (2020) and Zhao et al. (2008). One SR/MA (Lyu et al., 2020) was performed for subgroup analysis based on treatment duration. In Lyu et al. (2020), CHM was found to be better than WM in improving migraine duration at the end of treatment, while there was no significant difference at the end of follow-up.

##### 3.6.5.3 Chinese Herbal Medicines Plus Western Medicines vs. Western Medicines

Three SRs/MAs (Chen et al., 2015; Lei et al., 2020; Zhai et al., 2022) assessed migraine duration and showed that CHM plus WM could improve migraine duration compared with WM alone.

#### 3.6.6 Acute Medication Usage

Only one SR (Lyu et al., 2020) reported the use of acute medication. The meta-analysis showed that CHM was better than WM in terms of acute medication usage.

#### 3.6.7 Adverse Events

Adverse events were evaluated in eight SRs/MAs, with the main adverse events being digestive symptoms, including nausea, diarrhea, and ventosity. In general, these SRs/MAs showed that there was no difference between CHM and WM with regard to adverse events, except for two SRs/MAs which stated that the incidence of adverse events with CHM was lower than with WM.

#### 3.6.8 Summary of Quality of Evidence

A total of 69 outcomes were assessed using the GRADE system. The results showed that the quality of evidence was moderate in 1 study, low in 13, and very low in 55. No outcomes were assessed as high quality. The downgrading factors were risk of bias (*n* = 69), inconsistency (*n* = 40), imprecision (*n* = 39), and publication bias (*n* = 61). The details of the quality of evidence are shown in Table 7

**TABLE 7:** GRADE profile for the quality of evidence.

Outcomes	Study ID	N/n	Pooled effect size	95% CI	I^2^%	Risk of bias	Consistency	Directness	Precision	Publication bias	Quality of evidence
Migraine days											
CHM vs. PC	Chen, 2015	3/122	MD = −1.25	(−1.91,−0.60)^*^	55	−1^a^	−1^c^	0	−1^e^	−1^f^	Very low
	Shan, 2018	3/347	MD = −0.74	(−1.30,−0.18)	0	−1^a^	0	0	−1^e^	−1^f^	Very low
CHM vs. WM	Shan, 2018	2/295	MD = −0.50	(−0.80,−0.20)	0	−1^a^	0	0	−1^e^	−1^f^	Very low
	Lyu, 2020^(1)^	4/446	MD = −1.65	(−3.85,0.54)	96	−1^a^	−2^d^	0	0	−1^f^	Very low
	Lyu, 2020	3/386	MD = −2.18	(−5.08,0.72)	97	−1^a^	−2^d^	0	−1^e^	−1^f^	Very low
	Lai, 2017	2/187	MD = −2.77	(−7.26,1.72)	99	−1^a^	−2^d^	0	−1^e^	−1^f^	Very low
Responder rate											
CHM vs. WM	Lyu, 2020	5/467	RR = 1.37	(1.23,1.52)^*^	0	−1^a^	0	0	0	−1^f^	Low
CHM vs. PC	Xiao, 2015	5/470	RR = 4.63	(2.74,7.80)^*^	0	−1^a^	0	0	0	−1^f^	Low
Pain intensity											
CHM vs. PC	Chen, 2015	3/122	MD = −1.11	(−1.43,−0.79)^*^	0	−1^a^	0	0	−1^e^	−1^f^	Very low
	Xiao, 2015	2/90	SMD = −1.33	(−1.79,−0.87)^*^	0	−1^a^	0	0	−1^e^	−1^f^	Very low
	Shan, 2018	3/236	SMD = −0.71	(−0.98,−0.43)^*^	0	−1^a^	0	0	−1^e^	−1^f^	Very low
CHM vs. WM	Shan, 2018	12/1036	SMD = −0.66	(−0.84,−0.47)^*^	51	−1^a^	−1^c^	0	0	0	Low
	Lai, 2017	16/1227	MD = −0.98	(−1.37,−0.59)^*^	93	−1^a^	−2^d^	0	0	−1^f^	Very low
	Zhai, 2022	2/108	MD = −0.44	(−2.11,1.22)	94	−1^a^	−2^d^	0	−1^e^	−1^f^	Very low
CHM + WM vs.WM	Lei, 2020	3/270	MD = −0.70	(−0.81,−0.59)^*^	0	−1^a^	0	0	−1^e^	−1^f^	Very low
	Zhai, 2022	4/301	SMD = −1.24	(−1.91,−0.56)^*^	85	−1^a^	−2^d^	0	−1^e^	−1^f^	Very low
VAS											
CHM vs. WM	Liu, 2018	3/226	MD = −1.59	(−2.13,−1.06)^*^	88	−1^a^	−2^d^	0	0	−1^f^	Very low
	Wu, 2020	3/267	MD = −1.83	(−2.69,−0.97)^*^	87	−1^a^	−2^d^	0	−1^e^	−1^f^	Very low
	Lyu, 2020^(1)^	14/1038	MD = −1.04	(−1.67,−0.40)^*^	96	−1^a^	−2^d^	0	0	0	Very low
	Lyu, 2020^(2)^	2/163	MD = −1.56	(−3.73,0.61)	96	−1^a^	−2^d^	0	−1^e^	−1^f^	Very low
CHM + WM vs.WM	Lei, 2020	8/741	MD = −1.59	(−2.13,−1.06)^*^	88	−1^a^	−2^d^	0	0	−1^f^	Very low
	Tian, 2018	2/138	MD = −1.10	(−1.25,−0.95)^*^	92	−2^b^	−2^d^	0	−1^e^	−1^f^	Very low
	Sun, 2018	4/328	MD = −1.04	(−1.42,−0.66)^*^	52	−2^b^	−1^c^	0	−1^e^	−1^f^	Very low
	Yu, 2020	2/226	MD = −1.41	(−2.02,−0.81)^*^	80	−1^a^	−2^d^	0	−1^e^	−1^f^	Very low
	Chen, 2020	3/356	MD = −1.59	(−2.10,−1.09)^*^	89	−1^a^	−1^c^	0	−1^e^	−1^f^	Very low
	Li, 2020	2/226	MD = −1.41	(−2.02,−0.81)^*^	80	−1^a^	−2^d^	0	−1^e^	−1^f^	Very low
	Wang,2019	7/589	MD = −0.94	(−1.09,−0.80)^*^	24	−1^a^	0	0	0	−1^f^	Low
NRS											
CHM vs. WM	Yu, 2020	2/438	MD = −0.31	(−0.95,0.33)	55	−1^a^	−1^c^	0	0	−1^f^	Very low
Migraine frequency											
CHM vs. PC	Xiao, 2015	5/492	SMD = −0.54	(−0.72,−0.36)^*^	8	−1^a^	0	0	0	−1^f^	Low
	Chen, 2015	3/122	MD = −1.42	(−1.81,−1.03)^*^	96	−1^a^	−2^d^	0	−1^e^	−1^f^	Very low
	Yu, 2020	2/438	MD = −1.94	(−4.28,0.40)	83	−1^a^	−2^d^	0	0	−1^f^	Very low
	Shan, 2018	5/547	SMD = −0.65	(−0.93,−0.38)^*^	54	−1^a^	−1^c^	0	0	−1^f^	Very low
	Shan, 2018	9/1343	SMD = −1.05	(−1.28,−0.82)^*^	55	−1^a^	−1^c^	0	0	0	Low
CHM vs. WM	Zhao, 2008	2/158	MD = 0.93	(−1,2.86)	90	−1^a^	−2^d^	0	−1^e^	−1^f^	Very low
	Liu, 2018	2/210	MD = −1.40	(−1.49,−1.32)^*^	0	−1^a^	0	0	−1^e^	−1^f^	Very low
	Yu, 2020	2/120	MD = −1.50	(−1.81,−1.19)^*^	12	−1^a^	0	0	−1^e^	−1^f^	Very low
	Zhai, 2022	1/68	MD = −0.60	(−1.12,−0.08)^*^	NA	−1^a^	0	0	−1^e^	−1^f^	Very low
	Lyu, 2020^(1)^	21/1567	MD = −1.23	(−1.69,−0.76)^*^	97	−1^a^	−2^d^	0	0	0	Very low
	Lyu, 2020^(2)^	5/345	MD = −0.96	(−1.70,−0.21)^*^	96	−1^a^	−2^d^	0	−1^e^	−1^f^	Very low
	Wu, 2020	3/275	MD = −1.61	(−2.07,−1.14)^*^	85	−1^a^	−2^d^	0	−1^e^	−1^f^	Very low
	Lai, 2017	14/1174	MD = −0.96	(−1.25,−0.67)^*^	90	−1^a^	−2^d^	0	0	−1^f^	Very low
CHM + WM vs.WM	Tian, 2018	2/339	MD = −1.86	(−2.00,−1.71)^*^	99	−2^b^	−2^d^	0	−1^e^	−1^f^	Very low
	Lei, 2020^(3)^	2/180	MD = −1.39	(−1.83,−0.95)^*^	0	−1^a^	0	0	−1^e^	−1^f^	Very low
	Lei, 2020^(4)^	2/148	MD = −2.08	(−2.34,−1.82)^*^	0	−1^a^	0	0	−1^e^	−1^f^	Very low
	Zhai, 2022	3/261	MD = −0.95	(−1.61,−0.29)^*^	90	−1^a^	−2^d^	0	−1^e^	−1^f^	Very low
Migraine duration											
CHM vs. PC	Chen, 2015	3/122	MD = −1.32	(−1.99,−0.65)^*^	81	−1^a^	−2^d^	0	−1^e^	−1^f^	Very low
	Shan, 2018	5/565	SMD = −0.50	(−0.68,−0.32)	8	−1^a^	0	0	0	−1^f^	Low
CHM vs. WM	Shan, 2018	9/787	SMD = −1.05	(−1.28,−0.82)	0	−1^a^	−1^c^	0	0	0	Low
	Lyu, 2020^(1)^	20/1495	MD = −2.24	(−3.18,−1.30)^*^	92	−1^a^	−2^d^	0	0	0	Very low
	Lyu, 2020^(2)^	3/250	MD = −3.6	(−8.85,1.66)	97	−1^a^	−2^d^	0	0	−1^f^	Very low
	Zhao, 2008	2/158	MD = 1.33	(−0.87,3.52)	0	−1^a^	0	0	−1^e^	−1^f^	Very low
	Lai, 2017	9/638	MD = −1.66	(−2.61,−0.71)^*^	87	−1^a^	−2^d^	0	0	−1^f^	Very low
	Yu, 2020	2/210	MD = −1.5	(−3.06,0.07)	94	−1^a^	−2^d^	0	−1^e^	−1^f^	Very low
	Zhai, 2022	1/68	MD = −1.4	(−1.99,−0.81)^*^	NA	−1^a^	0	0	−1^e^	−1^f^	Very low
CHM + WM vs. WM	Lei, 2020	9/1075	SMD = −3.13	(−4.12,−2.15)^*^	97	−1^a^	−2^d^	0	0	−1^f^	Very low
	Chen, 2015	2/189	MD = −8.46	(−12.03,−4.88)^*^	0	−1^a^	0	0	−1^e^	−1^f^	Very low
	Zhai, 2022	2/161	MD = −0.87	(−1.41,−0.34)^*^	59	−1^a^	−1^c^	0	−1^e^	−1^f^	Very low
Acute medication usage											
CHM vs. WM	Lyu, 2020^(1)^	5/506	MD = −0.58	(−1.03,−0.13)	94	−1^a^	−2^d^	0	0	−1^f^	Very low
	Lyu, 2020^(2)^	4/446	MD = −0.69	(−1.22,−0.15)	96	−1^a^	−2^d^	0	0	−1^f^	Very low
Adverse events											
CHM vs. WM	Yu, 2020	2/210	RR = 0.59	(0.22,1.56)	0	−1^a^	0	0	−1^e^	−1^f^	Very low
	Wang,2019	10/1097	OR = 0.4	(0.19,0.84)	0	−1^a^	0	0	0	0	Moderate
	Zhai, 2022	3/179	RR = 0.28	(0.09,0.86)	0	−1^a^	0	0	−1^e^	−1^f^	Very low
	Lai, 2017	9/640	OR = 0.43	(0.23,0.82)	0	−1^a^	0	0	0	−1^f^	Low
CHM + WM vs. WM	Yu, (2020)	3/322	RR = 0.85	(0.29,2.47)	0	−1^a^	0	0	−1^e^	−1^f^	Very low
	Tian, 2018	7/755	RR = 0.39	(0.25,0.62)	0	−2^b^	0	0	0	0	Low
	Chen, 2020	5/538	OR = 0.59	(0.24,1.45)	0	−1^a^	0	0	0	−1^f^	Low
	Zhai, 2022	4/449	RR = 0.98	(0.56,1.70)	0	−1^a^	0	0	0	−1^f^	Low
	Li, 2020	3/322	OR = 0.84	(0.28,2.56)	0	−1^a^	0	0	−1^e^	−1^f^	Very low
	Wang,2019	7/547	OR = 1	(0.28,3.61)	0	−1^a^	0	0	0	-1^f^	Low

(1) At the end of treatment; (2) at the end of follow-up; (3) the unit is time/months; (4) the unit is time/days.

a. The included studies have a high risk of bias in terms of randomization, blinding, allocation concealment, completeness of data, or selective reporting; b. the included studies have two or more high risks of bias in terms of randomization, blinding, allocation concealment, completeness of data, or selective reporting; c. 50%≤I^2^<75%; d. 75%≤I^2^≤100%; e. small sample-size studies accounted for the majority; f. asymmetric funnel plot or less than nine studies.

## 4 Discussion

### 4.1 Main Findings

This study covered 16 SRs/MAs published between 2008 and 2022. Eleven SRs/MAs were published in Chinese and five (Xiao et al., 2015; Shan et al., 2018; Wang et al., 2019; Lyu et al., 2020; Wu et al., 2020) in English. The SRs/MAs that were published in English were more standardized in terms of methodological and reporting quality than the majority of those published in Chinese, particularly regarding the reporting of possible conflicts of interest. We established three main findings by evaluating the methodological and evidence quality of the SRs/MAs published to date.

First, for this overview we used a narrative description of the data synthesis used in these SRs/MAs, which showed that CHM plus WM had a favorable effect on migraines. In comparison, there was conflicting evidence for the effectiveness of using CHM alone. Twelve SRs/MAs investigated the efficacy of CHM alone as a migraine treatment and found that CHM was better than WM in improving responder rate and acute medication usage, and was superior to placebo in improving migraine days, responder rate, and migraine duration. However, there was insufficient evidence to verify the effectiveness of CHM in migraine treatment with regard to pain severity and migraine frequency. Our findings indicate that CHM appears to be safe for migraine treatment. According to the data synthesis of the included SR/MA outcomes, we found that total efficiency was the most extensively used outcome indicator in the SRs/MAs and the original RCTs. However, this outcome indicator was a composite index; the efficacy evaluation criteria were usually self-designed or referred to in other literature. Therefore, it is challenging for this indicator to accurately reflect the efficacy of a treatment (Zhang et al., 2020). Therefore, we did not evaluate total efficiency due to these factors.

Second, the methodological quality and reporting of the included SRs/MAs was extremely low. High-quality SRs/MAs could provide sufficient evidence for clinical decision-making. It was common for us to overlook the program registration in the 16 SRs/MAs, which could reduce a study’s rigor. None of the SRs/MAs provided a list of excluded studies or explained their exclusion, which might lead to selection bias. Also, commercially funded studies are more likely to achieve results that favor the funder’s product. However, none of these systematic evaluations, including the RCTs, reported funding sources, which might lead to publication bias.

Third, 69 outcomes were assessed using the GRADE system; this showed that the quality of pooled evidence in these SRs/MAs was deficient. Therefore, we should be cautious about drawing conclusions based on their results. The main downgrading factor was risk of bias. All of the SRs/MAs included original RCTs that were of low-quality, lacking detailed descriptions of randomization, allocation concealment, and blinding techniques. SRs/MAs of studies with small sample sizes are more likely to have publication bias (Guyatt et al., 2011), as most of the included SRs/MAs in our study did. High heterogeneity was also a major cause of lower levels of evidence quality in 13 SRs/MAs. Just four SRs/MAs explained and dealt with high heterogeneity; three of them (Lai, 2017; Lei et al., 2020; Yu, 2020) used subgroup analysis, and three of them (Lei et al., 2020; Yu, 2020; Zhai et al., 2022) used sensitivity analysis. By extracting the included SR outcomes, we found that the evaluation criteria for the outcomes of CHM for migraine were not uniform, especially for total efficiency rate and migraine frequency.

### 4.2 Comparison with Current Recommendations

A clinical practice guideline for treating migraines using CHM has recently been published in China, which recommends five classic formulas (Chuanxiong Chatiao powder, Chuanxiong Dingtong Yin, Sanpian Decoction, Xuefu Zhuyu decoction, and Tongqiao Huoxue decoction) and four Chinese patent medicines (Zhengtian pills, Toutongning capsules, Tongtian oral liquid, and Yangxue Qingnao granules/pills); these treatments, alone or in combination with WM, have an effect on migraine, but the quality of evidence is generally low (Xu et al., 2020). The findings of our study were consistent with these recommendations. Due to the generally low quality of evidence, recent international guidelines have not yet recommended herbal medicine for migraines (American Headache Society, 2018; Araki et al., 2019; Steiner et al., 2019). An overview evaluating the evidence from SRs/MAs of CAM therapy for the prevention and treatment of migraine headaches was recently published (Posadzki et al., 2015). The authors evaluated SRs/MAs of herbal medicines, including *Ginkgo biloba*, *Petasites hybridus*, and *Tanacetum parthenium* L, which differed from our inclusion criteria. Our study aimed to evaluate the evidence relating to Chinese herbal formulas. We conclude that the evidence for the effectiveness of herbal medicine is conflicting.

### 4.3 Limitations

Our investigation had several limitations. First, due to the small number of systematic evaluations of individual drugs for migraine, we did not evaluate individual drugs. Second, individual herbal medicine treatments varied throughout the included SRs/MAs, as did the efficacy criteria. As a result, we did not quantitatively pool the evidence of all outcomes and simply described the evidence of the included studies.

### 4.4 Implications for Future Studies

Our results provide valuable evidence of the definitive effectiveness of CHM in the treatment of migraines. We have developed the following recommendations for future studies. First, systematic evaluations should be established in accordance with the PRISMA guidelines (Page et al., 2021), to improve their methodological quality and render their evidence more convincing. Second, clinical studies should employ enhanced methodological quality, such as the proper application of randomization, allocation concealment, and blinding methods. Third, the design of the clinical study protocol is equally essential. Investigators should consult the most recent controlled trial guidelines for preventive treatment of chronic migraine in adults (Tassorelli et al., 2018). To select outcome indicators in systematic assessments or clinical research, the efficacy evaluation criteria of outcome indicators should be clarified, and composite indicators should be used sparingly (Zhang et al., 2020).

This overview suggests that CHM may be beneficial in improving migraines and can be used as a complementary therapy. However, due to the low quality of both methodology and evidence, we should treat the conclusions of included studies cautiously. Thus, future studies should focus on improving the methodological and reporting quality of SRs/MAs to make our conclusions more clinically applicable, and standardized clinical study designs should be employed to provide clinicians with reliable evidence.

## Abbreviations

CAM, complementary and alternative medicine; CGRP, calcitonin gene-related peptide; CHM, Chinese herbal medicine; CI, confidence interval; GRADE, Grading of Recommendations Assessment, Development and Evaluation; MA, meta-analysis; NRS, numerical rating scale; OR, odds ratio; PC, placebo-controlled; PRISMA, Preferred Reporting Items for Systematic Reviews and Meta-analyses; RR, risk ratio; SMD, standard mean difference; SR, systematic review; VAS, visual analog scale; WM, Western medicine; WMD, weighted mean difference.
